# *Bordetella pertussis* Commits Human Dendritic Cells to Promote a Th1/Th17 Response through the Activity of Adenylate Cyclase Toxin and MAPK-Pathways

**DOI:** 10.1371/journal.pone.0008734

**Published:** 2010-01-15

**Authors:** Giorgio Fedele, Fabiana Spensieri, Raffaella Palazzo, Maria Nasso, Gordon Yiu Chong Cheung, John Graham Coote, Clara Maria Ausiello

**Affiliations:** 1 Department of Infectious, Parasitic and Immune-Mediated Diseases, Istituto Superiore di Sanità, Rome, Italy; 2 Division of Infection and Immunity, Institute of Biomedical and Life Sciences, Glasgow Biomedical Research Centre, University of Glasgow, Glasgow, United Kingdom; National Institute for Infectious Diseases (INMI) L. Spallanzani, Italy

## Abstract

The complex pathology of *B. pertussis* infection is due to multiple virulence factors having disparate effects on different cell types. We focused our investigation on the ability of *B. pertussis* to modulate host immunity, in particular on the role played by adenylate cyclase toxin (CyaA), an important virulence factor of *B. pertussis*. As a tool, we used human monocyte derived dendritic cells (MDDC), an ex vivo model useful for the evaluation of the regulatory potential of DC on T cell immune responses. The work compared MDDC functions after encounter with wild-type *B. pertussis* (BpWT) or a mutant lacking CyaA (BpCyaA−), or the BpCyaA− strain supplemented with either the fully functional CyaA or a derivative, CyaA*, lacking adenylate cyclase activity. As a first step, MDDC maturation, cytokine production, and modulation of T helper cell polarization were evaluated. As a second step, engagement of Toll-like receptors (TLR) 2 and TLR4 by *B. pertussis* and the signaling events connected to this were analyzed. These approaches allowed us to demonstrate that CyaA expressed by *B. pertussis* strongly interferes with DC functions, by reducing the expression of phenotypic markers and immunomodulatory cytokines, and blocking IL-12p70 production. *B. pertussis*-treated MDDC promoted a mixed Th1/Th17 polarization, and the activity of CyaA altered the Th1/Th17 balance, enhancing Th17 and limiting Th1 expansion. We also demonstrated that Th1 effectors are induced by *B. pertussis*-MDDC in the absence of IL-12p70 through an ERK1/2 dependent mechanism, and that p38 MAPK is essential for MDDC-driven Th17 expansion. The data suggest that CyaA mediates an escape strategy for the bacterium, since it reduces Th1 immunity and increases Th17 responses thought to be responsible, when the response is exacerbated, for enhanced lung inflammation and injury.

## Introduction

*Bordetella pertussis* is a Gram-negative coccobacillus that causes whooping cough, a respiratory disease representing a severe, life-threatening illness in infants and children and a significant cause of morbidity in adolescents and adults [1]–[3]. The incidence of pertussis is increasing, even in countries with high vaccine coverage where immunity to pertussis is waning and a growing number of vaccinated adults have lost protection. Consequently, *Bordetella* infection is once again on the rise as a public health threat [2], [4]. Many aspects of pertussis pathogenesis and the mechanisms involved in protection from the disease are not fully understood, a situation complicated by the large number of virulence factors expressed by *B. pertussis* that exert disparate effects on different cell types [5], [6].

Adenylate cyclase toxin (CyaA) is secreted by *B. pertussis* and binds to the cell surface receptor CD11b/CD18 α_M_β2 integrin which is expressed on innate immune cells, including macrophages and dendritic cells (DC) [7]. Within cells, CyaA is able to disrupt host cellular functions by increasing the intracellular cyclic AMP (cAMP) concentration [8]–[10]. CyaA is able to suppress host antibactericidal activity by inhibiting chemotaxis, phagocytosis, superoxide production and expression of pro-inflammatory cytokines, such as TNF-α and IL-12p70, in monocyte/macrophages, neutrophils and DC [11]–[16], thereby promoting bacterial colonization and persistence [17], [18]. Moreover CyaA is also able to induce apoptosis in macrophages [19], [20].

DC are major players in innate and adaptive immunity, as they express repertoires of pathogen-recognition receptors that recognize specific molecular signatures of pathogens and trigger antimicrobial defense responses. Activation of DC leads to the production of cytokines and other immune mediators promoting the development of specific T helper (Th) responses that play a critical role in host protective immunity against infectious agents [21], [22]. We have previously shown that *B. pertussis* triggers maturation of human monocyte-derived (MD)DC that efficiently perform antigen presentation and, although unable to produce IL-12p70, drive the development of Th1 immunity [23]. In a subsequent study we demonstrated that IL-12p70 is inhibited through a block of IL-12p35 expression [15].

In this study we focused on the mechanisms that *B. pertussis* employs to escape host immune responses and the role played by CyaA. The experimental approach involved the analysis of T cell responses induced by human MDDC cultured with wild type *B. pertussis* (BpWT) or an isogenic mutant strain lacking CyaA, BpCyaA− [18], and by using fully functional recombinant CyaA or a derivative lacking adenylate cyclase activity, CyaA*, to complement the BpCyaA− strain [24], [25]. These approaches allowed us to demonstrate the induction by *B. pertussis* of MDDC promoting a mixed Th1/Th17 polarization and to clarify the role played by the p38 and ERK1/2 mitogen-activated protein kinases (MAPK) in this process. The data shed light on the role played by CyaA in subverting host immune responses by enhancing Th17 immunity and limiting the induction of Th1 effectors.

## Results

### *B. pertussis* CyaA Inhibits MDDC Maturation and Cytokine Production

After the encounter with a pathogen, the differentiation program of MDDC starts with the modification of surface markers expression [21], [22]. In preliminary experiments MDDC were treated with BpWT or with BpCyaA− and the ability of MDDC to internalize the two types of bacteria and the intracellular survival of internalized bacteria were measured, as reported in a previous study for BpWT [23]. The BpCyaA− strain behaved similarly to BpWT, both strains had a comparable low capability to be internalized by- and to survive in- MDDC (data not shown).

Phenotypic maturation of MDDC treated with BpWT or with BpCyaA− was then assessed. In order to restore adenylate cyclase activity to BpCyaA−, recombinant CyaA was simultaneously administered and a genetically inactivated toxin, lacking adenylate cyclase enzymatic activity, CyaA*, was used as a control. In previous studies we defined the optimal bacteria∶MDDC ratio to induce MDDC maturation and functions [23] and here, in preliminary experiments, we finely titrated CyaA and CyaA* doses in MDDC cultures. We found that a 50 ng/ml dose of CyaA was optimal to maximize the induction of intracellular cAMP and to minimize the induction of apoptosis (data not shown). Table 1 summarizes MDDC surface expression of the co-stimulatory molecule CD80 and the maturation markers CD83 and CD38 induced by the bacteria. Both *B. pertussis* strains induced significant up-regulation of phenotypic markers with respect to untreated MDDC. The BpCyaA−strain was able to induce significantly higher levels of CD80 and CD83 expression compared to BpWT strain (*p*<0.05), confirming and extending previous data [15], [23]. The expression of CD38, a newly described maturation marker involved in the regulation of IL-12p70 [26], [27], was also higher in BpCyaA− -treated MDDC compared to BpWT -treated MDDC (*p*<0.05). Expression of maturation markers declined to levels similar to those induced by BpWT when CyaA was added to MDDC cultured with BpCyaA−, but not when CyaA* was used (Table 1). No relevant modification of expression was found when the toxins were used alone to stimulate MDDC, indicating that any LPS contamination of the recombinant CyaA proteins was very low, confirming the results of the LAL test [24], [25], and not detectable even using MDDC maturation as a functional biological assay.

**Table 1:** MDDC phenotypical modulation by CyaA toxin after treatment with *B. pertussis* strains^a^.

Stimuli	CD80^b^ (MFI)	CD83^b^ (% of positive cells)	CD38^b^ (MFI)
BpWT	44±4 (2.3±0.2)^c^	39±6 (2.3±0.4)^c^	23±2 (2.1±0.2)^c^
BpCyaA−	76±12 (2.2±0.3)c,d,e	46±5 (2.6±0.8) c,d,e	42±5 (2.6±0.4)c,d,e
BpCyaA−+CyaA	41±10 (2.6±0.2)^c^	19±4 (2.4±0.1)^c^	19±4 (2.5±0.4)^c^
BpCyaA−+CyaA*	87±20 (2.4±0.3)^c^	40±9 (2.5±0.9)^c^	40±9 (2.6±0.8)^c^
none	20±2 (2.1±0.3)	4±1 (2.1±0.6)	11±2 (2.4±0.3)
CyaA	21±3 (2.5±0.1)	6±3 (2.0±0.2)	9±1 (2.2±0.1)
CyaA*	22±4 (2.2±0.2)	2±1 (2.7±0.7)	9±1 (2.1±0.3)

a MDDC were treated with indicated stimuli for 48 h and analyzed for indicated surface markers associated with the mature phenotype. Isotype-matched antibodies were used as negative controls and the values are shown in brackets.

b Mean value ± SE of 23 experiments is indicated.

c p<0.05 *vs* none;

d p<0.05 *vs* BpWT;

e p<0.05 *vs* BpCyaA−+CyaA.

These phenotypic data revealed that CyaA was responsible for the inhibition of CD80, CD83 and CD38 expression in *B. pertussis* -treated MDDC, thereby reducing the overall degree of DC maturation.

**Figure 1:**
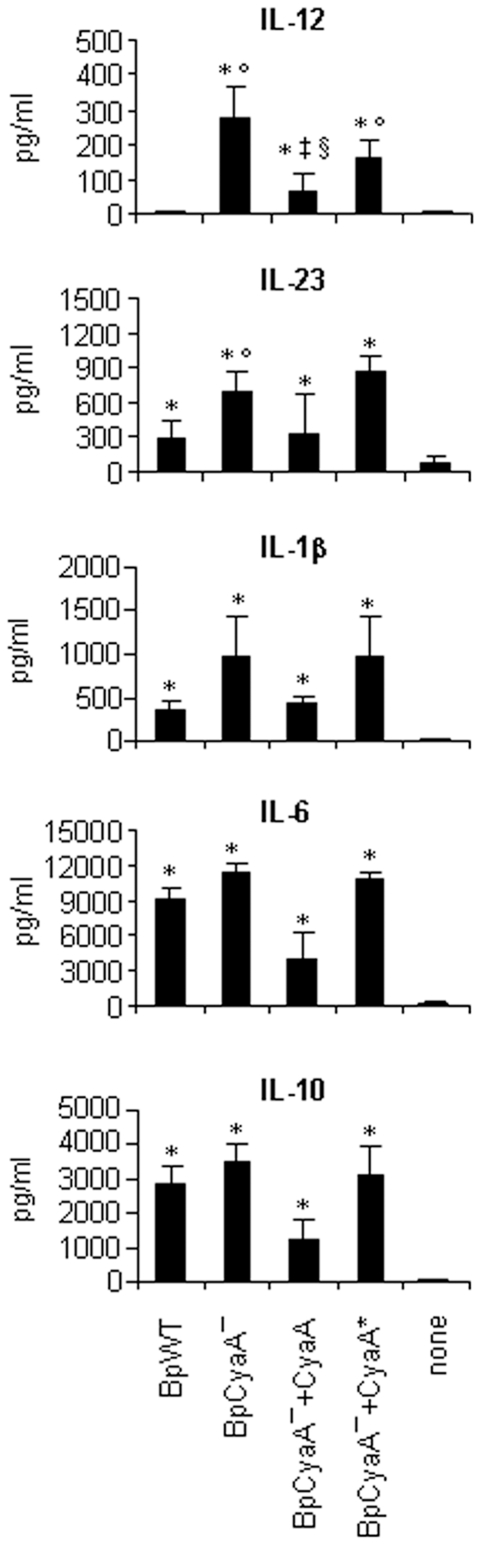
Cytokine production. MDDC were either untreated (none) or treated with BpWT or BpCyaA−, the latter in the presence or absence of CyaA and CyaA* (50 ng/ml ) for 48 h. IL-12p70, IL-23, IL-1β, IL-6 and IL-10 were assessed by ELISA. Values are expressed as mean ± SE from nineteen (IL-12p70), eleven (IL-10), six (IL-23, IL-1β and IL-6) independent experiments performed with MDDC obtained from different donors, and expressed as pg/ml of cytokine released. * *p*<0.05 *vs* none; °*p*<0.05 *vs* BpWT; ^‡^ *p*<0.05 *vs* BpCyaA−; ^§^ *p*<0.05 *vs* BpCyaA−+CyaA*.

DC maturation leads to the production of several regulatory cytokines pivotal in driving T helper (Th) cell polarization. Here, we analyzed the role of CyaA in modulation of cytokine production by MDDC in the presence of *B. pertussis*. BpWT strain was unable to induce IL-12p70 secretion, while BpCyaA− promoted the release of statistically significant levels of IL-12p70 (*P* = 0.01) (Figure 1), confirming previous data [15], [23]. In order to confirm that CyaA was responsible for IL-12p70 inhibition, MDDC were cultured with BpCyaA− in the presence of CyaA or CyaA*. Addition of CyaA strongly and significantly inhibited IL-12p70 production with respect to BpCyaA−- and to BpCyaA−+CyaA* -treated MDDC. CyaA* addition did not significantly affect IL-12p70 induced by BpCyaA− -treated MDDC (Figure 1).

The combination of IL-23, IL-6 and IL-1β has been shown to be crucial in driving the differentiation and expansion of Th17 cells, a recently described Th subset involved in infections and autoimmune disorders [28]–[30]. In this context, we found that *B. pertussis* induced IL-23 as well as IL-1β and IL-6 in MDDC (Figure 1). IL-23 levels induced by BpWT were markedly lower compared to those induced by BpCyaA− (*P* = 0.002). The addition of CyaA to BpCyaA− -treated MDDC significantly inhibited IL-23 production, while the addition of CyaA* was ineffective (Figure 1). Moreover, CyaA, either expressed by BpWT or exogenously added to BpCyaA−, was associated with reduced IL-1β and IL-6 production, although without statistically significant differences. Both *B. pertussis* strains were able to induce high levels of IL-10 and the addition of CyaA inhibited IL-10 induced by BpCyaA− (Figure 1).

When used alone, CyaA and CyaA* did not induce any of the cytokines tested with the exception of IL-23, produced at very low levels not significantly different than those from untreated MDDC, confirming again that any LPS contamination of the proteins was undetectable (data not shown).

### CyaA Modulates Th1/Th17 Polarization Induced by B. pertussis Treated MDDC

**Figure 2:**
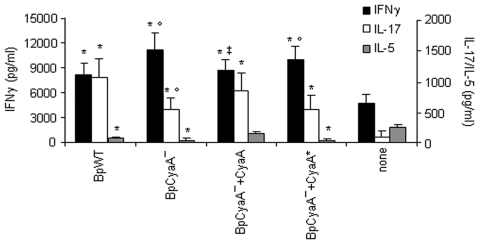
Th polarization. MDDC either untreated (none) or treated with BpWT or BpCyaA− for 48 h were co-cultured with purified T cells as described in Methods section. On day 12, supernatants were collected and secreted cytokines were measured by ELISA. Results are expressed as mean ± SE of eight independent experiments performed with MDDC and T cells obtained from different donors. * *p*<0.05 *vs* none; ° *p*<0.05 *vs* BpWT; ^‡^ *p*<0.05 *vs* BpCyaA−.

Considering the cytokine profile induced by *B. pertussis* in MDDC, we analyzed their capacity to polarize purified T lymphocytes, and, in particular, the possibility of Th17 induction/expansion. Figure 2 shows that BpWT -treated MDDC drove IFNγ- and IL-17 -producing Th effector cells. Remarkably, in view of the findings in the previous section, when T cells were co-cultured with BpCyaA− -treated MDDC, a significant decrease of IL-17 -producing cells was observed, along with a significant increase of IFNγ -producing cells. The addition of CyaA to BpCyaA− -treated MDDC reverted the polarization profile to that induced by BpWT and IL-17 -producing Th cells were enhanced, while those producing IFNγ significantly decreased. CyaA* did not cause any significant alteration of the polarization profile induced by BpCyaA− -treated MDDC (Figure 2). Th2 polarization, assessed by measuring IL-5 release, was significantly reduced when MDDC were treated with both *B. pertussis* strains, with respect to untreated MDDC.

### *B. pertussis* Triggers MyD88 - and Phosphatidyl Inositol 3 Kinase -Dependent Pathways in MDDC

**Figure 3:**
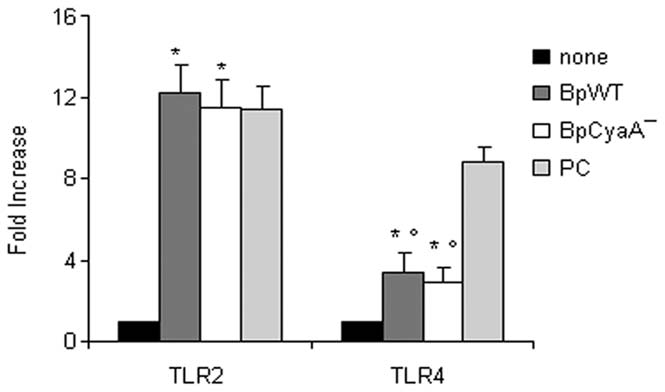
TLR4 and TLR2 activation. Triggering of TLR4 and TLR2 in transfected HEK293 cells. HEK293/TLR4/p-Nifty2-SEAP and HEK293/TLR2/p-Nifty2-SEAP cells were either untreated (none) or treated with BpWT or BpCyaA− for 16 h. Positive control (PC) for TLR4 stimulation was *E. coli* LPS (0.1 µg/ml) and positive control for TLR2 stimulation was Pam2CSK4 (0.1 µg/ml). SEAP activity in supernatants of cell cultures was measured. Data are reported as fold increase of SEAP activity over untreated values. Mean expression ± SE of ten independent experiments is indicated. * *p*<0.05 *vs* none; ° *p*<0.05 *vs* PC.

The activation of TLR4 and TLR2 by *B. pertussis* was investigated by using TLR2- and TLR4- expressing HEK-293 cells [31]. Figure 3 shows that BpWT and BpCyaA− were able to induce a potent activation of TLR2, at levels comparable to Pam2CSK4, the positive control. TLR4 signaling was also induced, but the levels of activation were statistically lower with respect to the ultra pure *E. coli* LPS, used as positive control.

The core component of TLR signaling is the activation of an IL-1-like pathway dependent upon the adapter MyD88, leading to phosphorylation of mitogen activated protein kinases (MAPK) such as p38, ERK1/2 and SAPK/JNK, and coupling to the activation of nuclear factor-κB (NF-κB) [32].

**Figure 4:**
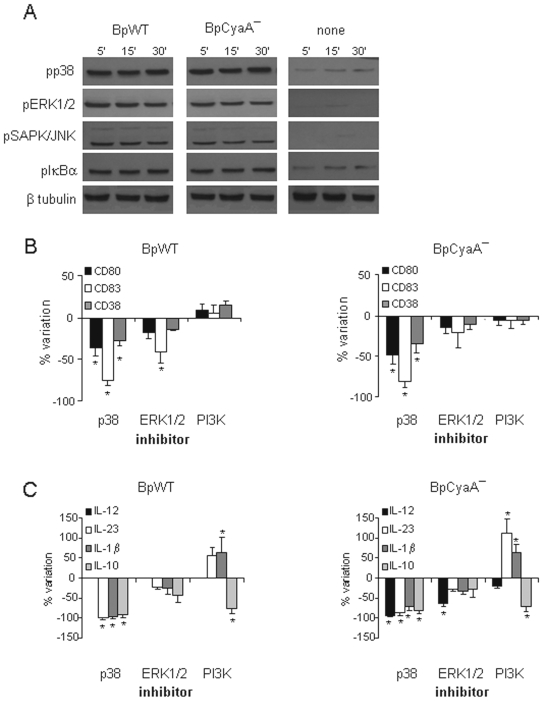
Analysis of MyD88 -dependent pathway induction. **A** MDDC were either untreated or treated with BpWT or BpCyaA−. Phosphorylation of p38, ERK1/2, SAP/JNK and IκB-α was determined at the indicated time-points by Western blot. A single gel was run and blotted to detect phosphorylated proteins and β tubulin to normalize the results. Data are from one representative out of four independent experiments performed with MDDC obtained from different donors. **B** MDDC were treated as in panel A, either in the absence or presence of p38 inhibitor (SB203580), ERK1/2 inhibitor (PD98059) or PI3K inhibitor (LY294002) for 48 h. Results of seven independent experiments performed with MDDC obtained from different donors are expressed as the percent of change of maturation markers (CD80, CD83, CD38) with respect to the corresponding stimulus in the absence of inhibitors. Mean ± SE of marker expression in MDDC not treated with inhibitors was for BpWT: CD80 (MFI) = 48±6; CD83 (%) = 23±6; CD38 (MFI) = 18±4; for BpCyaA−: CD80 (MFI) = 72±11; CD83 (%) = 36±7; CD38 (MFI) = 46±5. * *p*<0.05 *vs* control, calculated from the raw data. **C** MDDC were treated as in panels A and B. Results of ten (IL-12p70), eight (IL-23 and IL-10) and 5 (IL-1β) independent experiments performed with MDDC obtained from different donors, measured by ELISA, are expressed as the percent of change of cytokines with respect to the corresponding stimulus in the absence of inhibitors. Mean ± SE of cytokine production (pg/ml) in MDDC not treated with inhibitors was for BpWT: IL-12p70 = 0±0; IL-23 = 310±70; IL-1β  = 330±85; IL-10 = 2034±605; for BpCyaA−: IL-12p70 = 374±43; IL-23 = 672±140; IL-1β = 600±87; IL-10 = 2477±593. * *p*<0.05 *vs* control, calculated from the raw data.

Both *B. pertussis* strains induced the phosphorylation of p38, ERK1/2, SAPK/JNK, and IkBα, a process that allows the activation of the NF-κB complex, in MDDC within 5 minutes after treatment (Figure 4A). The induction of intracellular MAPK signaling was further analyzed using inhibitors specific for p38 (SB203580) and ERK1/2 (PD98059) and measuring phenotypic markers and cytokine release (Figure 4B–C). When MDDC were treated with either BpWT or BpCyaA−, p38 inhibitor induced a significant inhibition of all maturation markers, in particular CD83 (reduced by 90%) (Figure 4B), and almost completely abrogated IL-12p70, IL-23, IL-1β and IL-10 cytokine production (Figure 4C). The ERK1/2 inhibitor caused a significant reduction of CD83 maturation marker (by 45%) in the presence of BpWT with limited effects on cytokine production. When MDDC were treated with BpCyaA−, the ERK1/2 inhibitor had no significant influence on phenotypic maturation but elicited some reduction of cytokine production, particularly of IL-12p70 (inhibited by 70%).

These results demonstrated that *B. pertussis* exerted intracellular effects through both p38 and ERK1/2 activation, with the former having a prominent influence.

Phosphatidyl inositol 3 kinase (PI3K) is activated in response to various stimuli, including TLR2 engagement [33], thus, considering the strong involvement of TLR2 activation by *B. pertussis* (Figure 3), we investigated its influence in MDDC activation induced by *B. pertussis* by using LY294002, a selective inhibitor of this kinase. The data obtained showed an anti-inflammatory role of PI3K. While no major differences were observed in phenotypic maturation in the presence of either BpWT or BpCyaA− (Figure 4B), this inhibitor caused strong effects on cytokine production (Figure 4C), in particular a sharply increased level of IL-23 production (>100% in the case of BpCyaA−) and IL-1β (by more than 50% with both strains). Conversely, a clear reduction of IL-10 levels was obtained under these conditions.

### *B. pertussis* CyaA Blocks the Activation of the MyD88-Independent Pathway

**Figure 5:**
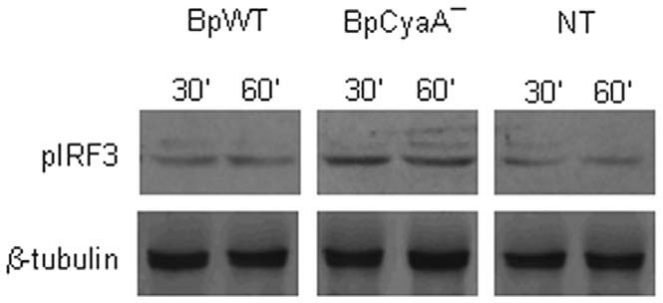
Analysis of MyD88-independent pathway induction. MDDC were either untreated (none) or treated with BpWT or BpCyaA−. Phosphorylation of IRF3 was determined at the indicated time-points by Western blot. Data are from one representative out of three independent experiments performed with MDDC obtained from different donors.

The TRIF-dependent signaling pathway downstream of TLR4 may be triggered independently of MyD88 and leads to the phosphorylation of interferon regulatory factor (IRF)3 and thereafter to the production of IFNβ and IFN-inducible genes, including IRF1 and IRF8. In a previous study we showed that BpWT blocks the expression of the p35 subunit of IL-12p70 through the inhibition of IRF1 and IRF8 expression due to CyaA-dependent intracellular cAMP accumulation [15]. Here, we found that BpCyaA− induced in MDDC a higher level of IRF3 phosphorylation compared to BpWT (Figure 5) and we confirmed that BpWT strongly inhibited the expression of IRF1 and IRF8. The addition of CyaA to BpCyaA−-treated MDDC was responsible for the inhibition of both IRF1 and IRF8 expression (not shown).

### p38 and ERK1/2 MAPK Regulate the Induction of Th17 and Th1 Polarization

**Figure 6:**
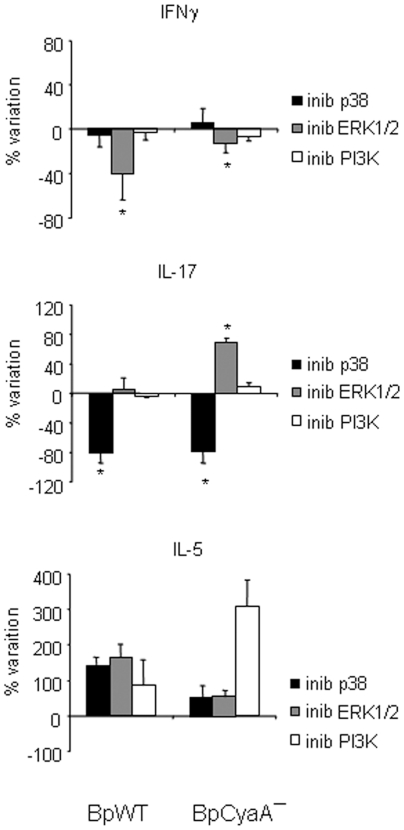
Effects of kinase inhibition on Th polarization. **A** MDDC, either untreated or treated with BpWT or BpCyaA− in the absence or presence of p38 inhibitor (SB203580), ERK1/2 inhibitor (PD98059) or PI3K inhibitor (LY294002) for 48 h, were co-cultured with purified T cells. On day 12, supernatants were collected and secreted cytokines were measured by ELISA. Results of four independent experiments performed with MDDC and T cells obtained from different donors are expressed as the percent of change of cytokines (IFNγ, IL-17 and IL-5) with respect to the corresponding stimulus in the absence of inhibitors. Mean ± SE of cytokine production (pg/ml) in MDDC not treated with inhibitors was for BpWT: IFNγ = 9013±1828; IL-17 = 900±68; IL-5 = 71±19; for BpCyaA−: IFNγ = 9834±1225; IL-17 = 423±94; IL-5 = 21±5. * *p*<0.05 *vs* control, calculated from the raw data.

To evaluate the contribution of activation pathways to the capacity of MDDC to polarize Th responses, we performed experiments where MDDC were treated with the two *Bordetella* strains in the presence of kinase inhibitors and then used to polarize purified T cells (Figure 6). When the capacity of *Bordetella* -treated MDDC to polarize a Th1 response was analyzed by measuring IFNγ, no specific effects were observed by using the p38 inhibitor, even though p38 inhibition caused a drastic reduction of all the cytokines measured in MDDC (Figure 4).

The use of ERK1/2 inhibitor clearly and significantly reduced IFNγ production by co-cultured T cells either when MDDC were treated with BpWT or BpCyaA−. These data indicate that ERK1/2 -dependent Th1 polarization is induced by *B. pertussis* independently of IL-12p70, which was not induced by BpWT in MDDC.

No specific effect was observed by the use of PI3K inhibitor.

When the capacity to polarize a Th17 response was analyzed after p38 inhibition, a clear and marked inhibition of IL-17 production by co-cultured T cells (more than 70%) was observed, either when MDDC were treated with BpWT or BpCyaA−.

When ERK1/2 was inhibited, a significant increase of IL-17 production by co-cultured T cells (by 80%) was observed when MDDC were treated with BpCyaA−.

PI3K inhibition did not cause any significant modification of IL-17 production when MDDC were treated with either BpWT or BpCyaA−.

The capacity to polarize a Th2 response was then analyzed and we found that p38, ERK1/2 and PI3K inhibition all caused an enhancement of IL-5 production induced by BpWT- and BpCyaA−-treated MDDC, although without any statistical significance as compared to untreated MDDC, suggesting a strong donor to donor variability in the production of this cytokine.

These results indicate a pivotal role for the ERK1/2 pathway in the induction of Th1 polarization and the p38 pathway in the induction of a Th17 response. All the pathways studied are implicated in the inhibition of the Th2 phenotype.

## Discussion

Several studies indicated that *B. pertussis* infection drives a Th1 immune response, mediated by IFNγ production, both in mice [6] and in humans [34], [35]. We recently demonstrated that *B. pertussis* induces IL-23 expression in human MDDC and promotes Th1 polarization in the absence of IL-12p70 [15], [23]. Here, we add new evidence showing that *B. pertussis* biased the host response toward a mixed Th1/Th17 phenotype through modulation of MDDC functions, and identified the CyaA and MAPKs as key players in this process. Indeed, CyaA limited Th1 and enhanced Th17 responses, and the p38 and ERK1/2 MAPK pathways were involved in the induction of Th17 and Th1 effectors, respectively.

*B. pertussis* triggers phenotypic maturation of MDDC [15], [23]. Here, we broadened our analysis to CD38. We confirmed that CD38 expression in MDDC was tightly linked to CD83 and IL-12p70 production [27]. Indeed, CD38, CD83 and IL-12p70 were all up-regulated when CyaA was absent (Table 1 and Figure 1). CD38 exerts pleiotropic activities in immune cells and particularly in DC [36]. Indeed, besides being functionally involved in CD83 expression and IL-12 p70 induction [27], CD38 also ensures efficient chemotaxis and transendothelial migration driven by CC chemokine ligand 21 (CCL21) [26]. Thus, the down regulation exerted by CyaA on CD38 functions can be considered as a possible escape mechanism adopted by the bacterium to hamper the host immune response.

Endogenous CyaA expressed by BpWT, or exogenously added to BpCyaA− -treated MDDC, limited the production of the cytokines analyzed. In particular, IL-23 and IL-1β were strongly reduced and IL-12p70 completely abrogated. MDDC treated with BpWT induced a Th1 polarized response, characterized by the production of high IFNγ levels (Figure 2). These results were in agreement with our previous studies [15], [23] and confirmed that *B. pertussis* induces in MDDC the expression of Th1 promoting factor(s) other than IL-12p70. As expected, BpCyaA− -treated MDDC were stronger inducers of Th1 polarization than BpWT -treated MDDC (Figure 2), due presumably to the action of IL-12p70.

The relevance of IL-17 activities in response to *Bordetella* infection has been pointed out in mice [37]–[39], and in humans [40], [41]. The present study demonstrated that Th17 polarization was induced *ex vivo* by *B. pertussis* -treated MDDC. Interestingly, IL-17 induced in T cells by BpWT -treated MDDC was greater than that from BpCyaA− -treated MDDC, indicating that CyaA promoted IL-17 production by T cells. However, the capacity to induce a stronger Th17 polarization appeared to be unrelated to the levels of Th17 promoting cytokines, such as IL-23, IL-1β, and IL-6, that were expressed at higher levels by BpCyaA− -treated MDDC. Instead, the regulatory activity of IL-12p70, induced in MDDC in the absence of active CyaA, may play a prominent role in inhibiting Th17 polarization. This would be in agreement with studies showing that IL-12p70 is detrimental for the expansion of Th17 cells [42]. Overall, the ex vivo polarization studies showed that CyaA was ultimately responsible for enhancing Th17 and reducing Th1 immunity induced by *B. pertussis*.

TLR4 signaling mediated by *B. pertussis* was reported to activate IL-10 production in mice [43] and to be involved in whole cell vaccine-induced protection through Th1/Th17 induction [39]. Here we demonstrated a low intensity TLR4 and a strong TLR2 activation by *B. pertussis* (Figure 3). TLR4 engagement by *B. pertussis*, although at low levels, was sufficient to activate the MyD88-independent pathway, as testified by phosphorylation of IRF3 (Figure 5), a link between TLR4 activation and the induction of interferon inducible IRF1 and IRF8 necessary for MyD88-independent IL-12p70 induction [44], [45]. Both IRF3 phosphorylation and IRF1/IRF8 mRNA transcription were better promoted by BpCyaA− than BpWT indicating an inhibition of MyD88-independent pathway by CyaA, that might represent a further mechanisms adopted by *B. pertussis* to dampen the host immune responses.

The use of MAPK inhibitors demonstrated a decisive role for p38 in sustaining phenotypic maturation and cytokine production, while ERK1/2 activity was involved in IL-12p70 production in BpCyaA− -treated MDDC (Figure 4B, C). Th1 induction, in the form of IFNγ production by BpWT -treated MDDC still occurred after p38 inhibition (Figure 6), thus developing independently of IL-12p70, which is no longer produced in these conditions (Figure 4C). These data, together with the observation that BpWT -treated MDDC promoted a Th1 expansion in the absence of IL-12p70, indicated the presence of a non-canonical Th1 driving signal induced by *B. pertussis*. The fact that inhibition of ERK1/2 pathway in MDDC resulted in significant decrease of IFNγ production by T cells (Figure 6), suggested that the Th1 driving signal was dependent on ERK1/2 activity.

Thus, two different pathways appeared involved in the Th1 polarization. ERK1/2 signals might be responsible of IFNγ induction by BpWT -treated MDDC in the absence IL-12p70, while in BpCyaA− -treated MDDC p38-dependent IL-12p70 production enhances the Th1 profile.

Concerning Th17 polarization, it was dramatically reduced by the p38 inhibitor (Figure 6). This result may reflect the strong reduction of IL-1β and IL-23 production caused by p38 inhibition in MDDC (Figure 4C).

ERK1/2 inhibition induced a significant increase of IL-17 production promoted by MDDC treated with BpCyaA− (Figure 6), likely due to a sharp inhibition of IL-12p70 production accompanied by a slight reduction of IL-23 and IL-1β in BpCyaA− -treated MDDC, suggesting a negative regulatory role of IL-12p70 in the development of Th17 immunity [42].

Inhibition of PI3K activity in MDDC increased production of IL-23 and IL-1β together with a concomitant reduction of IL-10, suggesting an anti-inflammatory role of this kinase involved in the regulation of innate responses to microbial pathogens [33]. However, PI3K inhibition did not cause appreciable changes in Th polarization, apart from an increase in IL-5 levels with BpCyaA− -treated MDDC (Figure 6).

The fact that *B. pertussis* is able to potentiate the Th17 response, even in the absence of CyaA, might be linked to the presence of pertussis toxin. We have recently demonstrated that genetically detoxified Pertussis toxin (dPT) -treated MDDC drive a mixed Th1/Th17 polarization [41]. Like *B. pertussis*, dPT activates the p38 and ERK1/2 pathways in MDDC, regulators of cytokine expression, and the PI3K pathway, crucial in limiting IL-23 and IL-1β and enhancing IL-10 production. A major difference from the data reported here was in T cell polarization by dPT-treated MDDC, where p38 inhibition markedly reduced the production of IFNγ by co-cultured T cells, reflecting the induction of the canonical p38 -dependent and IL-12p70 -mediated Th1 polarization. The differences observed in the activation pathway in MDDC/T cells in *B. pertussis* and dPT are not surprising considering the complexity of the interaction exerted by the bacterium upon MDDC activation.

Previous observations have shown that *B. pertussis* and *B. bronchiseptica* lacking CyaA caused a diminished inflammation pathology in mice, due to a reduced ability to recruit leucocytes, primarily neutrophils, suggesting that infiltrating cells are responsible for lung injury [46], [47]. It is now possible to re-interpret these data in the light of the results of this study. Here we have shown that CyaA ablation resulted in a reduced capacity to promote expansion of IL-17 producing effectors, and it is well known that the over-production of IL-17 fosters neutrophil recruitment to the site of inflammation [48].

In the light of the results depicted here, it is possible to speculate that CyaA mediates an escape strategy for the bacterium since it reduces Th1 immunity and increases the Th17 responses thought to be responsible, when the response is exacerbated, of enhanced lung inflammation and injury.

## Materials and Methods

### Ethic Statement

This study was conducted according to the principles expressed in the Declaration of Helsinki. All blood donors provided written informed consent for the collection of samples and subsequent analysis and the blood samples were processed anonymously. The study was approved by the Review Board of the University La Sapienza, Rome, Italy.

### Reagents

Recombinant *Bordetella pertussis* CyaA and CyaA* were expressed and purified as described elsewhere [24], [25]. Polymyxin B and Brefeldin A were purchased from Sigma Chemicals (St. Louis, MO). Purified *E. coli* LPS, synthetic bacterial lipoprotein S-[2,3-bis(palmitoloxy)-(2RS)-propyl]-[R]-cysteinyl-[S]-seryl-[S]-lysyl-[S]-lysyl-[S]-lysyl-[S]-lysine X 3CF3COOH (Pam2CSK4), p44-p42 ERK1/2 inhibitor PD98059, p38 MAPK inhibitor SB203580, PI3K inhibitor LY294002 were from Cayla - InvivoGen Europe (Toulouse, France). Human rGM-CSF and rIL-4 were from R&D Systems (Minneapolis, MN). rIL-2 was obtained from Roche (Basel, Switzerland). Fluorochrome-conjugated anti-human CD3, CD1a, CD14, CD38, CD80, CD83 and appropriated mouse isotype-matched mAbs were from BD Biosciences Europe (Erembodegem, Belgium). Rabbit polyclonal IgG anti-p-p44/42 mitogen-activated protein kinases (MAPK) (Thr202/Tyr204, ERK1/2), anti-p-p38 MAPK (Thr180/Tyr182), anti-p-JNK/Stress Activated Protein Kinase (SAPK) (Thr183/Tyr185), anti-p-I kappa B alpha (IκBα) (Ser32), anti-p-IRF3 Ab were from Cell Signaling Technology, Inc. (Danvers, MA). Mouse anti-β tubulin was from Invitrogen (Paisley, UK).

### Bacterial Strains and Growth Conditions

*B. pertussis* strains: Bp18323 (BpWT) (ATCC reference strain 97-97) and its isogenic Bp18HS19 mutant (BpCyaA−) [15], [18] were inoculated onto charcoal agar plates supplemented with 10% of sheep blood (Oxoid, Basingstoke, UK) and grown at 37°C for 72 h to visualize hemolysis and plated again on charcoal agar for 48 h at 37°C. Bacteria were then collected and re-suspended in 5 ml of phosphate buffered saline (PBS). Bacterial concentration was estimated by measuring the optical density at 600 nm and the suspension adjusted to a final concentration of 10^9^ CFU/ml. The bacterial concentration was then checked retrospectively by CFU evaluation of the final bacteria suspension.

### Purification and Culture of MDDC

Human monocytes were purified from peripheral blood of healthy blood donors (Courtesy of Dr. Ferrazza, “Centro Trasfusionale Policlinico Umberto I”, University La Sapienza, Rome, Italy) and cultured in RPMI 1640 (GIBCO, Invitrogen), supplemented with heat-inactivated 10% LPS-screened FCS, 1 mM sodium pyruvate, 0.1 mM nonessential amino acids, 2 mM L-glutamine, 25 mM HEPES (N-2-hydroxyethylpiprazin-N'-2-ethansulfonic acid), 100 U/ml penicillin, 100 µg/ml streptomycin, all from Hyclone Laboratories and 0.05 mM 2-ME (Sigma) (hereafter defined as complete medium) in the presence of GM-CSF (25 ng/ml) and IL-4 (25 ng/ml). After 6 days, immature MDDC were washed and analyzed by cytofluorometric analysis for CD1a, CD14 and CD38 expression. Where indicated, MDDC were treated with ERK1/2 inhibitor PD98059 (100 µM), p38 inhibitor SB203580 (20 µM) or PI3K inhibitor LY294002 (5 µM ) for 1 h prior to *B. pertussis* treatment. *E. coli* LPS (100 ng/ml) was used as positive stimulus to induce MDDC maturation.

MDDC (10^6^ cell/ml) were re-suspended in complete medium without penicillin and streptomycin (hereafter defined as Bp medium), and treated with *B. pertussis* cells as described elsewhere [23]. After 2h, cells were extensively washed in the presence of polymyxin B (5 µg/ml) and incubated at 37°C, 5% CO_2_ for 48 h in Bp medium with 100 U/ml penicillin and 100 µg/ml streptomycin added. In a previous study we tested several bacterium-to-cell ratios, and the 100∶1 was chosen as optimal ratio on the basis of bacterial ability to induce MDDC maturation without affecting viability [23].

Where indicated, recombinant CyaA (50 ng/ml) or CyaA* (50 ng/ml) was added at optimal dosage (determined in preliminary experiments) to MDDC cultures, alone or immediately after BpCyaA− treatment.

After 48 h, treated MDDC were harvested for immunophenotypic analysis and supernatants for cytokine measurement by ELISA.

### Cell Lines

Human epithelial kidney (HEK)-293 cells stably transfected with human TLR4, MD2 and CD14 (HEK/TLR4) or human TLR2 (HEK/TLR2) were purchased from InvivoGen; The HEK/TLR clones were grown in standard Dulbecco's modified Eagle's medium (DMEM) (GIBCO, Invitrogen) with heat-inactivated 10% LPS-screened fetal calf serum (FCS) (LAL<1 ng/ml LPS) supplemented with 1 mM sodium pyruvate, 0.1 mM non-essential amino acids, 2 mM L-glutamine, all from Hyclone Laboratories (Logan, OH), and normocin™ (100 µg/ml, InvivoGen), in a 5% saturated CO_2_ atmosphere at 37°C. HEK/TLR4 culture medium was supplemented with 0.2 mM Blasticidin (InvivoGen) and HygroGold™ (50 µg/ml, InvivoGen). HEK/TLR2 culture medium was supplemented with 0.6 mM G418 Sulfate (InvivoGen). HEK293/TLR cells were transfected with a plasmid encoding secreted alkaline phosphatase (SEAP) (pNifty2-SEAP, InvivoGen) as previously described [31].

### TLR Signaling Assay

The induction of TLR signaling in HEK/TLR/p-Nifty2-SEAP clones was assessed by measuring SEAP activity using QUANTI-Blue™ colorimetric assay (InvivoGen). Briefly, HEK/TLR/p-Nifty2-SEAP cells with specific antibiotics added, as specified before, were seeded into 24-well plates at a density of 2×10^5^ cells in 0.5 ml HEK medium per well for 48 h and then cells were either untreated (none), treated for 16 h with *B. pertussis* at 100∶1 bacterium-cell ratio (the optimal ratio to induce maximal TLR4 or TLR2 signaling without affecting cell viability), ultrapure LPS from *E. coli* or Pam2CSK4. Supernatants (10 µl) were then transferred to a 96-well plate and incubated at 37°C with QUANTI-Blue™ (200 µl). SEAP activity was measured by reading optical density at 655 nm with a 3550-UV Microplate Reader (BioRad, Philadelphia, PA). Data are reported as the fold induction of SEAP activity over untreated controls.

### Isolation and Polarization of T Lymphocytes

T cells were purified from peripheral blood mononuclear cells by negative sorting with magnetic beads (Pan T-cell Kit, Milteniy Biotec, Auburn, CA). Purity of cell preparations was assessed by cytofluorometric staining [23]. T cells (0.5×10^6^) were cultured in complete medium in 24-well plates (Costar) in the presence of MDDC (0.5×10^5^). On day 5, IL-2 (50 U/ml) was added to the cultures. On day 12, supernatants were harvested for cytokine measurement.

### Immunophenotypic Analysis

Cells were washed and re-suspended in PBS containing 3% FBS and 0.09% NaN_3_, then incubated with a panel of fluorochrome-conjugated mAbs (obtained from BD Biosciences, San Jose, CA) specific for MDDC: anti-CD14, CD1a, CD80, CD83 and CD38; or specific for T cells: anti-CD3. Isotype-matched antibodies were used as negative control. Cells were analyzed with a FACScan (BD Biosciences). Fluorescence data are reported as percentage of positive cells when treatment induces the expression of the marker in cells that were negative; median fluorescence intensity (MFI) was used when treatment increased the expression of the marker in cells that were already positive.

### Determination of Cytokine Levels by ELISA

To measure cytokine production, MDDC were cultured in the presence of the indicated stimuli in 12 ml tubes (Falcon, Becton Dickinson, Lincoln Park, NJ) at 37°C, 5% CO_2_. Supernatants were collected after 48 h, and IL-10, IL-12p70, IL-23, IL-1β and IL-6 production was assessed by ELISA (Quantikine, R&D Systems, Minneapolis, MN) and IL-23 by Bender MedSystem (Burlingame, CA) with a sensitivity of 1.0 pg/ml for IL-1β, 0.7 pg/ml for IL-6, 3.9 pg/ml for IL-10, 5.0 pg/ml for IL-12p70 and 20.0 pg/ml for IL-23. Optical density obtained was measured with a 3550-UV Microplate Reader (BioRad Philadelphia, PA) at 450 nm.

Cytokines in the supernatants from polarized T cells were assayed by ELISA specific for IFNγ, IL-5 and IL-17 (Quantikine, R&D Systems). The lower detection limits were 8.0 pg/ml for IFNγ, 3.0 pg/ml for IL-5, 15.0 pg/ml for IL-17.

### mRNA Cytokine Expression by TaqMan Real-Time Reverse Transcriptase-PCR Aanalysis

To measure cytokine mRNA expression, TaqMan Real-time Reverse Transcriptase-PCR (RT-PCR) analysis was used (Applied biosystems, Foster City, CA). Total RNA was extracted from MDDC at different time points and reverse transcription was carried out as previously described [15]. TaqMan assays were performed according to manufacturer's instructions with an ABI 7700 thermocycler (Applied biosystems). PCR was performed, amplifying the target cDNA (IRF1 and IRF8) transcripts and the β-actin cDNA as endogenous control. Data obtained were analyzed with PE Relative Quantification software of Applied Biosystems. Specific mRNA transcript levels were expressed as fold increase compared to basal conditions.

### Western Blot Analysis

MDDC were starved by culturing overnight in culture medium supplemented with 1% LPS-screened FCS. Cells were then stimulated with *B. pertussis* at different time points and lysed in RIPA buffer as previously described [40]. Immunoreactive protein was detected by incubating blots with anti-phosphorylated proteins (or anti-unphosphorylated β-tubulin as control) overnight at 4°C. Blots were washed in Tris-buffered saline 0.1% Tween-20, incubated with horse-radish-peroxidase (HRP)-conjugated goat anti-rabbit IgG (Bio-Rad Laboratories, Hercules, CA) (to reveal phosphorylated proteins) or HRP-conjugated goat anti-mouse IgG (GE Healthcare) (to reveal control proteins) developed with the enhanced chemiluminescence (ECL) reagents from Pierce (Rockford, IL).

### Statistical Analysis

Statistical descriptive analyses were carried out using the SPSS Inc (Chicago, IL) statistical package. Differences between mean values were assessed by two-tailed Student's *t* test and were statistically significant for P values <0.05.
